# Genomic evidence refutes the hypothesis that the Bornean banteng is a distinct species

**DOI:** 10.1186/s12862-022-02062-1

**Published:** 2022-09-20

**Authors:** Xin Sun, Marta Maria Ciucani, Jacob Agerbo Rasmussen, M. Thomas P. Gilbert, Mikkel-Holger S. Sinding

**Affiliations:** 1 University Museum, NTNU, 7491 Trondheim, Norway; 2 Center for Evolutionary Hologenomics, Globe Institute, Faculty of Health and Medical Sciences, University of Copenhagen, 1353 Copenhagen, Denmark; 3 Section for Computational and RNA Biology, Department of Biology, University of Copenhagen, 1350 Copenhagen, Denmark

**Keywords:** Bornean banteng, Genomics, Phylogeny, Taxonomy

## Abstract

The banteng (*Bos javanicus*) is an endangered species within the wild Asian *Bos* complex, that has traditionally been subdivided into three geographically isolated subspecies based on (i) mainland Southeast Asia (*B. j. birmanicus*), (ii) Java (*B. j. javanicus*), and (iii) Borneo (*B. j. lowi*). However, analysis of a single Bornean banteng mitochondrial genome generated through a genome skimming approach was used to suggest that it may actually represent a distinct species (Ishige et al. in Mitochondrial DNA A DNA Mapp Seq Anal 27(4):2453–4. http://doi.org/10.3109/19401736.2015.1033694 , 2016). To explore this hypothesis further, we leveraged on the GenBank (NCBI) raw read sequencing data originally used to construct the mitochondrial genome and reconstructed its nuclear genome at low (0.2×) coverage. When analysed in the context of nuclear genomic data representing a broad reference panel of Asian *Bos* species, we find the Bornean banteng affiliates strongly with the Javan banteng, in contradiction to the expectation if the separate species hypothesis was correct. Thus, despite the Bornean banteng’s unusual mitochondrial lineage, we argue there is no genomic evidence that the Bornean banteng is a distinct species.

## Introduction

The wild Asian *Bos* complex consists of three recognised species in Southeast Asia, the banteng (*B. javanicus*), the gaur (*Bos gaurus*), and the kouprey (*Bos sauveli*) that likely went extinct in the twentieth century [1]. The subspecies level structure and diversity of this group has previously only been characterised using mitochondrial DNA, revealing clear paraphyletic structure, with the Bornean banteng mitochondrial lineage nesting inside the diversity of the gaur [2]. Based on this observation, it has been suggested that the Bornean banteng may not be a banteng at all, but rather a distinct species in the wild Asian *Bos* complex [2]. Mitochondrial DNA based phylogenetic signals are, however, not always concordant with those generated from autosomal nuclear DNA [3]. Therefore, we elected to leverage on the fact that the mitochondrial genome in question was generated through genome skimming of shotgun sequence generated from the tooth of a banteng bull collected in 2010 in Malua, Sabah, Borneo, thus un-analysed nuclear DNA data also exists for the specimen in the public record. We therefore mapped this unused fraction of the original genome skim data to the water buffalo reference genome, enabling us to recover the autosomal genome of the Bornean banteng at an average depth of 0.2×. In this communication we put this partial genome into a phylogenetic context.

## Materials and methods

We downloaded the Borneo banteng shotgun genome skim dataset (NCBI: SRA: DRA00172) generated by [2], computationally processed it through the pipeline recently used to generate and investigate the first kouprey genomes [4] along with wild Asian *Bos* genome wide variation. Specifically, reads were mapped using the PALEOMIX pipeline v1.2.13.2 [5]. Low quality and missing bases were trimmed from the reads with default settings, and adaptors, dimers and sequences shorter than 25 bp were removed using AdapterRemoval 2.2.4 [6]. To avoid biases associated with mapping to an ingroup [7] in downstream ancestry analyses, retained reads were mapped against scaffolds above 50.000 bp of the outgroup—de novo Water buffalo (*Bubalus bubalis*) UMD_CASPUR_WB_2.0 [8]. The reads were then aligned to the reference using bwa-aln v0.7.16a algorithm [9] including minimum base mapping quality to optimise the initial coverage. Filters targeting mapping and base quality were added at a later stage as appropriate in the specific analysis. Next, Picard MarkDuplicates v2.18.0 (http://broadinstitute.github.io/picard/) was used to filter for PCR and optical duplicates. Finally, GATK v4.1.0.0 [10, 11] was used to perform the indel realignment step with no external indel database. All analyses were performed using a set of reference alignments published in [4]. The specific analyses performed was a mitochondrial neighbour-joining phylogeny made using MEGA 10 [12] with 500 bootstraps. ANGSD v0.921 [13] was used to estimate the genotype likelihood at the varying sites for scaffolds with a length above 10 kbp. The resulting output in beagle format was subsequently used as input in PCAngsd v0.973 to perform a genotype likelihood based principal component analysis (PCA). The covariance matrix was used as input in Rstudio [14] to calculate eigenstrat and eigenvalues and plot the components using ggplot2 [15]. We randomly selected 1000 sequences of 5000 bp to construct gene trees using RAxML [16], which were then used to build the species tree. A nuclear genome phylogeny (species tree) was made in ASTRAL-III [17], with DiscoVista being implemented to evaluate the support of alternative topologies [18]. All analyses were performed using the same parameters as detailed in [4].

## Results and discussion

**Fig. 1:**
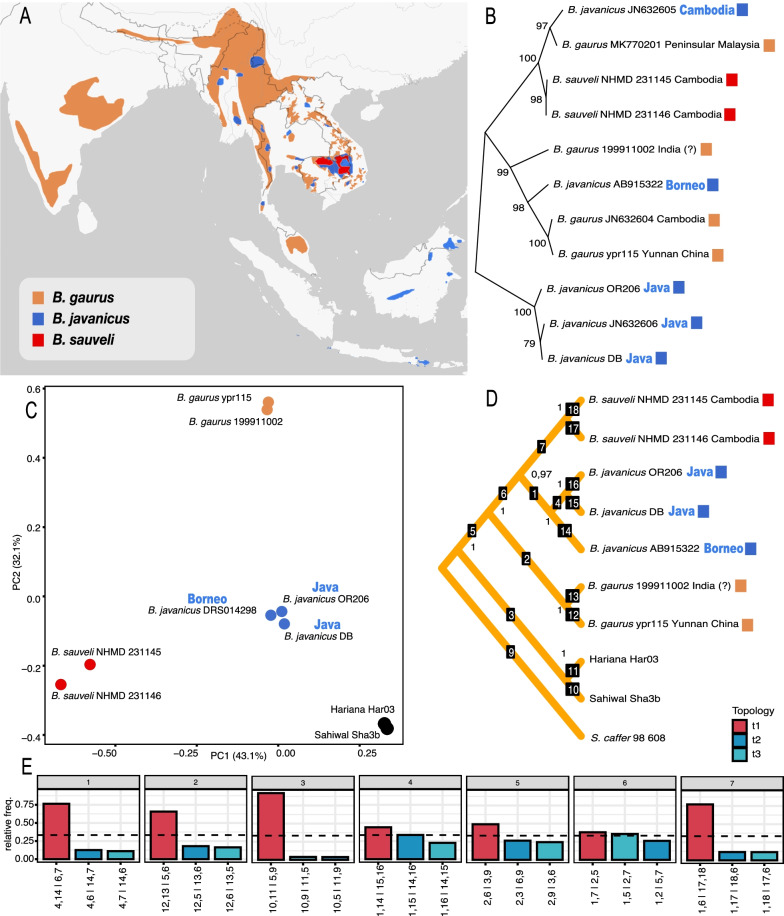
Distribution of wild Asian *Bos* and genetic results. **A** Current geographic range based on IUCN data, *B. sauveli* (possibly extinct), *B. gaurus* (extant), and *B. javanicus* (extant, possibly extant and possibly extinct) [1, 21, 22]. **B** Mitochondrial phylogeny rooted to the Javan banteng clade. Bootstrap support is given at the base of nodes. **C** PCA of full nuclear genomes. **D** Nuclear genome phylogeny estimated by ASTRAL-III. The tree is rooted to the African buffalo (*Syncerus caffer*). Numbers placed at branch nodes represent clade supports expressed in posterior probabilities and computed by RAxML-ng using 100 replicates in Astral-III. **E** Display of quartet frequencies of the three possible configurations of internal branches in the nuclear phylogeny, when evaluating clades as an underlying unrooted tree. Red bars show the topscore configuration presented in the phylogeny (**D**), whereas the two blue bars show alternative configurations. Alternative tree configurations are labelled corresponding to branch IDs in (**D**). The dotted line indicates a level of a one-third bipartition for every quartet, which is the threshold frequency of a true bipartition [23]

Today the species level distribution of banteng (Fig. 1A) exists as three geographically isolated groups (one on the Southeast Asian mainland, a second on Java and a third on Borneo), which correspond with the three described banteng subspecies. Consistent with the prior mitochondrial-based analysis of the Bornean subspecies, we replicate the paraphyletic mitochondrial structure of banteng (Fig. 1B), finding that the Bornean banteng lineage is nested within that of the gaur. To explore the nuclear genomic affiliation of the Bornean banteng, we generated a PCA based on two gaur, two kouprey, two zebu cattle (*Bos taurus indicus*) and two captive banteng of Javan origin (based on both zoo records, also supported by the mitochondrial data). The results show clustering of all the distinct species, including notably, the Bornean banteng with the Javan bantengs (Fig. 1C). To further evaluate a phylogenetic affiliation at the autosomal level, we used the same genomic dataset to create an ASTRAL-III nuclear phylogeny, allowing scaffold-based evaluation of the support for alternative topologies using DiscoVista. Again all species form individual clusters, supported by the highest posterior probability of 1, with the Bornean banteng robustly placed on a branch as sister to the two Javan banteng (Fig. 1D). Additionally, despite the limited amount of data available for performing the analyses (constrained by the, 0.2× coverage of the Bornean banteng), the DiscoVista results (Fig. 1E) replicate previous findings of extensive incomplete lineage sorting (ILS) at the diversification of wild Asian *Bos* [4]. This is compatible with a polytomic origin of the three species [4], we hypothesise may explain the inconsistency of the mitochondrial versus the nuclear phylogenetic affiliation. A comparable scenario is seen in ILS in bison, where European wisent (*Bison bonasus*) and American bison (*Bison bison*) carry highly divergent mitochondrial lineages [19]. As a result American bison mitochondria form a sister lineage to yak (*Bos mutus*), while wisent mitochondria form a sister lineage to cattle (*Bos taurus*). However at autosomal level European wisent and American bison are unambiguously related and monophyletic.

Should ILS not be the explanation, one alternative explanation could be that low levels of hybridisation might have led to fixation of an exotic mitochondrial lineage in either (or both) of the insular banteng groups. Potentially low levels of gaur admixture might have occured during the formation of the Bornean banteng subspecies either prior to, or during, the isolation of Borneo after the last glacial maximum [20]. Alternatively we speculate that the Javan banteng could potentially have inherited the unique mitochondrial lineage from an unknown, now extinct population. These are all interesting scenarios, that research including ancient genomes may clarify in the future.

However, regardless of the origin of the banteng’s paraphyletic mitochondrial structure, the 0.2× nuclear genome clearly supports affiliation of the Bornean banteng with the banteng species. Despite this, we highlight that these results by no means decrease the conservation value of the Bornean banteng, but rather allow focus and coherence by clarifying species affiliation. According to IUCN, the current banteng species population is decreasing in size, and consists of between 4000 and 8000 mature individuals that are isolated, dispersed and as a whole endangered [21]. What fraction of these are Bornean is largely unknown—while probably no more than 350 individuals live in Sabah, only very small fragmented populations exist in Kalimantan, and likely very few—if any—persist in Sarawak [21]. Ultimately therefore we hope that our genomic characterization of the Bornean banteng population might contribute to both future attempts to clarify its evolutionary history, as well as guide future conservation efforts.

## Data Availability

All data analysed in this study is publicly available and previously published, the NCBI affiliation of each specimen is given in Fig. 1.
